# Targeting Mitochondria for Cancer Treatment

**DOI:** 10.3390/pharmaceutics16040444

**Published:** 2024-03-23

**Authors:** Ljubava D. Zorova, Polina A. Abramicheva, Nadezda V. Andrianova, Valentina A. Babenko, Savva D. Zorov, Irina B. Pevzner, Vasily A. Popkov, Dmitry S. Semenovich, Elmira I. Yakupova, Denis N. Silachev, Egor Y. Plotnikov, Gennady T. Sukhikh, Dmitry B. Zorov

**Affiliations:** 1 A.N. Belozersky Research Institute of Physico-Chemical Biology, Lomonosov Moscow State University, 119991 Moscow, Russia; lju_2003@list.ru (L.D.Z.); polinaabr92@mail.ru (P.A.A.); nucleus-90@yandex.ru (V.A.B.); zorov@inbox.ru (S.D.Z.); irinapevzner@mail.ru (I.B.P.); popkov.vas@gmail.com (V.A.P.); 7emenovich@gmail.com (D.S.S.); elmira.yaku@gmail.com (E.I.Y.); silachevdn@belozersky.msu.ru (D.N.S.); plotnikov@belozersky.msu.ru (E.Y.P.); 2 V.I. Kulakov National Medical Research Center of Obstetrics, Gynecology and Perinatology, 117997 Moscow, Russia; 3 Faculty of Bioengineering and Bioinformatics, Lomonosov Moscow State University, 119991 Moscow, Russia

**Keywords:** mitochondria, cancer, tumor, energetics, hypoxia, fumarate reductase, ROS, TSPO, peripheral benzodiazepine receptor, rhodoquinone

## Abstract

There is an increasing accumulation of data on the exceptional importance of mitochondria in the occurrence and treatment of cancer, and in all lines of evidence for such participation, there are both energetic and non-bioenergetic functional features of mitochondria. This analytical review examines three specific features of adaptive mitochondrial changes in several malignant tumors. The first feature is characteristic of solid tumors, whose cells are forced to rebuild their energetics due to the absence of oxygen, namely, to activate the fumarate reductase pathway instead of the traditional succinate oxidase pathway that exists in aerobic conditions. For such a restructuring, the presence of a low-potential quinone is necessary, which cannot ensure the conventional conversion of succinate into fumarate but rather enables the reverse reaction, that is, the conversion of fumarate into succinate. In this scenario, complex I becomes the only generator of energy in mitochondria. The second feature is the increased proliferation in aggressive tumors of the so-called mitochondrial (peripheral) benzodiazepine receptor, also called translocator protein (TSPO) residing in the outer mitochondrial membrane, the function of which in oncogenic transformation stays mysterious. The third feature of tumor cells is the enhanced retention of certain molecules, in particular mitochondrially directed cations similar to rhodamine 123, which allows for the selective accumulation of anticancer drugs in mitochondria. These three features of mitochondria can be targets for the development of an anti-cancer strategy.

## 1. Introduction

The role of mitochondria in oncogenesis has been scrutinized in a huge number of experimental and analytical works. Here we will limit ourselves to analyzing those problems relevant to this topic that rarely become the subject of consideration, but their significance and understanding are no less important than other, very often considered, aspects of mitochondrial involvement in the occurrence and therapy of cancer, which will only very briefly be considered in our analysis.

## 2. Major Energetics-Related and Non-Related Changes in Cancer Cells

To more fully assess the role of mitochondria in the occurrence and treatment of any pathology, including cancer, it is necessary to move away from the magnetism of a one-sided view of the mitochondria as exclusively an energy machine in the cell. The role of non-energetic functions of mitochondria is wide and includes several unique synthetic functions that guide the processes of cellular signaling aimed at energetics itself, thermogenesis, cell death, detoxification, etc. [1,2]. From these alternative functions of mitochondria comes a greater importance in the regulation of oncogenesis than directly from the oxidation associated with phosphorylation, that is, the synthesis of ATP. The synthesis of ATP by the mitochondria of tumor cells can be strongly inhibited, but mitochondria are still present in these cells, which indicates that the ATP synthesizing function of mitochondria is not dominant in the tumor cell and that mitochondria are needed to perform functions other than ATP synthesis.

The most widely discussed change in energy metabolism in tumor cells is an increased glycolytic capacity [3,4,5,6,7,8,9,10,11,12,13]. Theoretically, this could be caused by the fact that the growth of tumor cells outruns the development of the circulatory system and limited angiogenesis, which ensures the growth of blood vessels that bring the oxidative substrates and oxygen necessary to perform oxidative phosphorylation into the tumor tissue (especially in the stage of a solid tumor). As a result, hypoxia becomes an attribute of a developing tumor, which requires the inclusion of an alternative, even less effective, mechanism of energy formation, that is, glycolysis. However, this evolutionarily developed mechanism of energy generation in a tumor cell exists a priori and is ahead of the onset of real hypoxia; the predominance of the glycolytic pathway over oxidative phosphorylation is observed even in the presence of a high concentration of O_2_ [3,4,5,6,7,8,9,10,11,12,13]. In some cases, as in hepatoma cells, the rate of glycolysis is ten times higher than in normal hepatocytes [5,13]. An increase in the expression of glycolysis enzymes and glucose transporters in different tumor cells has been proven [14].

It has been suggested that this increase in glycolytic flow is a metabolic strategy of tumor cells to ensure survival and growth in environments with low O_2_ concentrations [12]. Several mechanisms of glycolysis enhancement in tumor cells have been developed and documented. It should be emphasized that there is no reason to automatically apply the mechanisms described below to all cancer cells; each particular line of tumor cells has its own combination of mechanisms and degree of expression to enhance glycolysis.

At first glance, it seems that the glycolytic energy metabolism of a cancer cell can be a principal target, using glycolysis inhibitors to kill the cell. However, given the understanding that the tumor cell forms a microenvironment designed to fight the cancer cell in which immune cells are present [15], which also become glycolytic when activated, the use of glycolysis inhibitors to kill the cancer cell is not appropriate.

One of the most important problems in cancer therapy is the extremely high heterogeneity of the cells in a tumor. The metabolism in one tumor may be different from that in another, though they may be similar in nature. As a result, the possibility of a universal approach to therapy seems remote. There are significant differences even in the behavior of particular glycolytic enzymes in different tumors, so the targets for exposure require an individual approach. On the other hand, it should be noted that the generally accepted opinion that all cancer cells are glycolytic is dogmatic; analysis shows that some cancer cells can derive energy from glycolysis, and some (for example, lung carcinoma, breast cancer, melanoma, sarcoma, ovarian and uterus carcinoma, etc.) primarily use oxidative phosphorylation for this purpose. As such, the above statement, which has been made by Warburg in 1956 [16] and his followers (e.g., see [3,4,17]), is not strictly correct.

One of the proven ways to suppress respiration is the activation of kinase, which can phosphorylate a key mitochondrial enzyme on which the flow of reduced equivalents into the Krebs cycle depends, namely pyruvate dehydrogenase. This leads to its inhibition and reduces the oxidation of pyruvate in the Krebs cycle, thereby increasing the formation of lactate from pyruvate [18,19]. A powerful inducer of this kinase is HIF-1α, which rapidly degrades in normoxia; in hypoxia, it is stabilized and begins to work as a transcription factor for several genes, including those that activate the expression of glycolytic enzymes [20,21]. However, in a tumor environment, which may not necessarily be hypoxic, a state of pseudohypoxia may occur with the same result as in hypoxia, due to increased lactate generation during anaerobic glycolysis, which also stabilizes HIF-1α [22]. A similar effect of HIF-1α stabilization is observed with the inhibition of succinate dehydrogenase, accompanied by the accumulation of succinate (the latter was confirmed with the use of mitochondria-targeted vitamin E [23], which has an anti-cancer effect).

Several mechanisms have been described that explain the activation of glycolysis in several tumors, most of which are localized in the mitochondria. Among them are an increase in the expression of glycolytic enzymes and glucose transporters, decreased expression of mitochondrial enzymes of oxidative metabolism, a decrease in the number of mitochondria in the cell, inhibition of oxidative phosphorylation by glycolysis (the Crabtree effect), an increase in the amount of protein inhibitor (IF1) in mitochondria, and higher sensitivity of mitochondrial DNA to oxidative stress [14].

Ultimately, it becomes clear that targeting the mitochondria of a cancer cell is one of the main strategic ways to combat this global problem. We will consider several mitochondrial targets that are not often amenable to analysis. One intrinsic feature of cancer cells is their excessive generation of reactive oxygen species (ROS) [24,25,26]. It is known that, in low concentrations, ROS perform vital signaling functions, but elevated levels of ROS cause oxidative modifications that can be harmful or even fatal [27]. However, these concentration dependences are extremely specific and, in several cells that specialize in the generation of ROS for the elimination of pathogens (such as immune cells or alveolar lung epithelial cells), there is an elevated protection against ROS due to the inherent high concentration of ROS inside the cell and in its environment. It is this seemingly paradoxical situation that has been noted in the cells of the naked mole rat, known for its extraordinary longevity, which ultimately provides the cells with high resistance to the damaging effects of ROS [28,29,30,31,32]. The logical conclusion from these facts is to exploit this property for therapeutic purposes by changing the cellular redox status [33].

## 3. Fumarate Reductase

It is clear that under hypoxic conditions (when the oxygen concentration is below cytochrome oxidase affinity), the electron transfer chain in mitochondria cannot function because there is no final electron acceptor. Under normoxic conditions, dioxygen interacts with cytochrome oxidase, receiving four electrons from it, followed by an interaction with protons to form two molecules of water. In the absence of oxygen, cytochrome oxidase does not function, meaning that both the coupling site associated with cytochrome oxidase and the coupling site upstream of cytochrome oxidase (bc1 complex) lose their ability to provide energy for ATP synthesis.

As a result, the mitochondrial system under hypoxia adapts to this situation with two main goals: to ensure at least a small production of ATP due to the functioning of complex I and to prevent the formation of an excessive amount of NADH.

The main reformatting of energetics in hypoxia occurs at the level of functioning of the Krebs cycle, which can only work under conditions of constant outflow of NADH formed in the Krebs cycle, and which cannot be performed with a non-functional respiratory chain. The evolutionary solution to these issues was to use the available components but with certain additions. If, under normoxic conditions in the Krebs cycle, succinate is converted into fumarate catalyzed by succinate dehydrogenase, then, under hypoxic conditions in anaerobic bacteria and eukaryotes (e.g., in nematodes such as *Ascaris lumbricoides* or *Caenorhabditis elegans*), a reverse reaction of fumarate formation into succinate occurs. Such catalysis is provided by fumarate reductase, which is different from succinate dehydrogenase [34].

**Figure 1:**
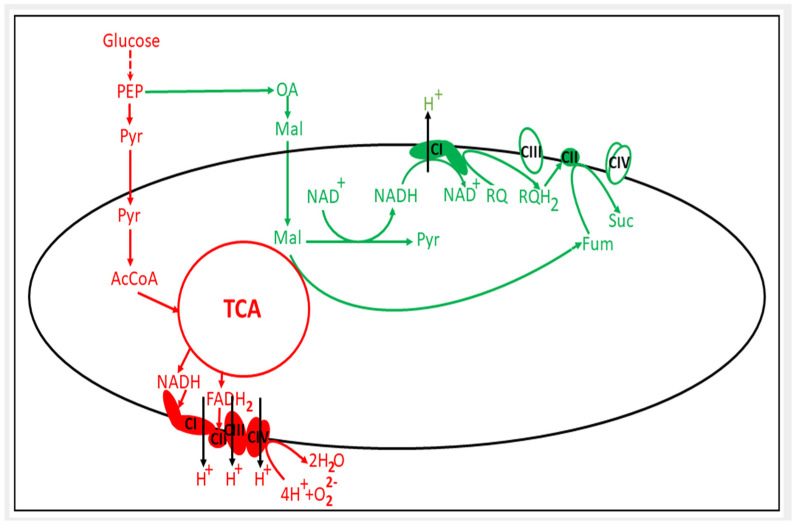
Changes in mitochondrial energetic metabolism under hypoxia. In red, the aerobic energy metabolism is shown; in green, the adaptive pathway of mitochondrial energetics to hypoxic conditions is schematically depicted. PEP, phosphoenolpyruvate; Pyr, pyruvate; OA, oxaloacetate; Mal, malate; RQ, rhodoquinone; Suc, succinate; Fum, fumarate; TCA, tricarboxylic acid cycle; CI, CII, CIII and CIV are respiratory complexes I–IV.

It must be understood that, for thermodynamic reasons, it is not very easy to support this reaction (fumarate–succinate); due to that canonical quinone (ubiquinone), which is reduced by the complex I, with the redox potential (E = +110 mV) supporting a direct succinate/fumarate reaction. Evolutionarily, to ensure fumarate respiration, the hypoxic system acquired another but low potential quinone (rhodoquinone, E = −63 mV), which cannot drive the succinate–fumarate reaction, but can drive the reverse reaction (fumarate–succinate) [33]. Under normoxic conditions, succinate dehydrogenase oxidizes succinate to form fumarate and reduced FAD, followed by the reduction of ubiquinone, which further participates in the respiratory chain. On the other hand, under hypoxic conditions, complex I reduces low-potential rhodoquinone instead of ubiquinone. The latter interacts with the succinate dehydrogenase complex, reducing FAD and triggering the fumarate-succinate reaction [34,35] (Figure 1).

It should be noted that the activation of the fumarate reductase pathway in hypoxia, which clear yielding a low extraction of energy from mitochondrial activity, is accompanied by a certain increase in the membrane potential of mitochondria. This indicates that under hypoxic conditions, both the reversal of ATP synthase activity and the coupled activation of fumarate reductase is the driving force for building the mitochondrial membrane potential [36,37]. This has been shown to be both a critical factor for and mandatory attribute of mitochondrial functioning [38]. 

It remains unclear whether there is an identical mechanism in the cancer cell (Figure 1). Unfortunately, it must be stated that in cancer cells, although the presence of a fumarate reductase pathway [31,38,39,40,41,42] leading to an increased level of succinate [43,44,45] has been proven, the question as to whether cancer cells endowed with rhodoquinone or other low-potential quinone has not yet been clarified. In addition to the two representatives of nematodes cited, there is evidence of the presence of this quinone in *M. edulis* (mussel), *C. angulata* (oyster), *L. stagnalis* (snail), *F. hepatica* (liver fluke), and *D. viviparus* (worm). As Chinopoulos wrote in his paper, “fumarate reductase remains to be discovered in mammalian mitochondria” [45].

We must admit that helminths switch from ubiquinone to rhodoquinone synthesis principally via changes in the alternative splicing of COQ-2, gene coding polyprenyltransferase COQ-2 [46], that involved in the kynurenine pathway of rhodoquinone synthesis [47,48]. The relevance of this system to solid cancer cells has not yet been elucidated. 

Whatever the mechanism of existing fumarate reductase activity in cancer cells [33,39,40,41,42,43,44], it should be accompanied by the absence of succinate oxidase activity and the reversal of succinate dehydrogenase activity (succinate: quinone oxidoreductase). Note that even though they functionally carry out two reversible reactions, succinate dehydrogenase and fumarate reductase both represent two separate oligomeric enzymes [49]. Quite recently, a reasonable idea was presented, suggesting that fumarate reductase is a common name for different representatives of the fumarate reductase family, covering different representatives of prokaryotes and eukaryotes whose structure and functionality were adapted to different environments, which means a high plasticity of the organization of such a mechanism for switching energetics under a decrease in ambient oxygen [50]. Details of the fumarate reductase pathway observed in cancer cells have not yet been properly developed. 

Mitochondrial Complex II can allow both succinate dehydrogenase and fumarate reductase activity to operate in both direct (succinate-fumarate) or reversed (fumarate-succinate) modes depending on metabolic shift of compromised bioenergetics and environmental factors [14,43,44,51,52]. One of the most remarkable features of such a rewiring is that while the production of ROS is insignificant in a direct reaction, it sharply increases with the activation of the reverse pathway [53,54]. ROS, in this case, originate from a FAD binding area and not from the electrons of Fe-S centers or bound quinones. Hypoxia, being either physical (a drop in ambient O_2_) or chemical (inhibition of cytochrome oxidase by some drugs), leads to the reversal of the work of Complex II and the reduction of the ubiquinone in the Q-cycle. This yields an increase in the probability of one-electron leakage from the reduced ubiquinone to available O_2_, the concentration of which, although small under conditions of physical hypoxia, is insufficient to obtain four electrons from cytochrome oxidase with subsequent formation of water [53,54,55,56]. The product of single electron transfer, superoxide anion radical is a member of the ROS family and can be the initial component for production of other ROS. 

Paradoxically, on the one hand, tumor mitochondria produce more ROS, which are an important factor for ensuring the characteristic increased proliferation of cancer cells [24]. On the other hand, one of the strategies for combating cancer cells is the use of so-called mitocans (i.e., mitochondria directed anticancer drugs [57,58]), one of the mechanisms of action of which is in enhanced generation of ROS in tumor cells, triggering their apoptosis [59,60,61]. Therefore, the ROS strategy to cope with cancer cells can take two forms, either to reduce the formation of ROS in them to prevent their proliferation or to sharply increase their level in cells to trigger apoptosis.

Considering the knowledge of the mechanism that provides a cell resistance to hypoxia, several targets immediately arise to destroy this mechanism and with it the host cell. These targets are: 1. fumarate reductase (it is required to inhibit it); 2. rhodoquinone or other quinones supporting fumarate-succinate conversion if found in tumor cells (to slow down their synthesis as follows for rhodoquinone from [47,48]); 3. succinate (see a strategy to alleviate its effects in the next chapter); and 4. ROS (use antioxidants, including mitochondria-directed ones or respiratory inhibitors [62,63,64,65], to neutralize ROS or instead to activate their production [24,59,60,61]). Whether all or some lines of strategy can be applied to a cancer cell must be developed and evaluated. In addition to these four options, and based on their specific anti-cancer activities, mitocans have been classified and organized in groups [58]. 

## 4. Succinate and Cancer

The question of the fate of succinate, formed because of a decrease in the activity of succinate dehydrogenase (conversion of succinate to fumarate) or an increase in activity leading to the activation of a reverse reaction in which fumarate is converted to succinate, is quite reasonable. In the first scenario, inhibition of succinate dehydrogenase activity may occur, in particular, because of mutations in succinate dehydrogenase subunits (SDHB or SDHD) characteristic of hereditary tumors and sporadic cancer, or with a decrease in the expression of the same subunits, which is a hallmark of various types of cancer [66,67,68,69,70]. Another mechanism for increasing succinate levels is the process of glutaminolysis [71]. 

In any of these scenarios, succinate accumulates in the tumor cell. In principle, the accumulation of succinate inside the cell (its content levels in the cell can rise from micromolar to millimolar levels) is not harmful for the cell. When working with isolated mitochondria, the usual working succinate concentration is 5–10 mM. However, it is at such succinate concentrations that a process is observed, namely the reverse transfer of electrons to the components of complex I with enhanced generation of reactive oxygen species, and most mitochondrial experts recognize that this is the most powerful source of ROS in mitochondria [72,73]. We will return to this issue below, but we must note that this process is so far limited to in vitro data and has not yet been detected in cells.

**Figure 2:**
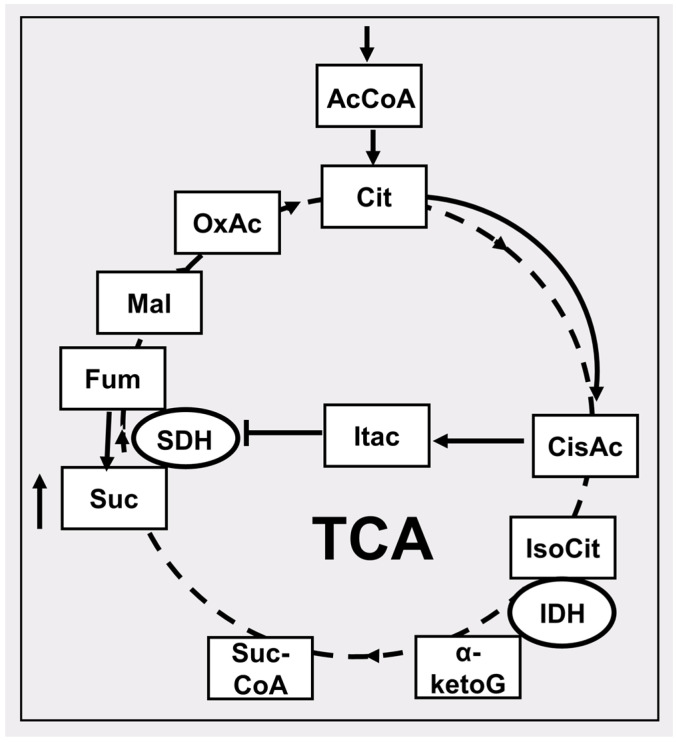
Particular features of the tricarboxylic acid cycle (TCA) in conditions of limited access to oxygen associated with the accumulation of succinate. The dotted lines show the reactions of the conventional course of the Krebs cycle under aerobic conditions. Solid lines show the course of reactions under hypoxic conditions leading to the accumulation of succinate. AcCoA, acetyl CoA; Cit, citrate; cisAc, cis-aconitate; Itac, itaconate; IsoCit, isocitrate; IDH, isocitrate dehydrogenase; α-ketoG, α-ketoglutarate; SucCoA, succinateCoA; Suc, succinate; SDH, succinate dehydrogenase; Fum, fumarate; Mal, malate, OxAc, oxaloacetate. The up arrow near the succinate box indicates an increase in its level.

There is another mechanism for increasing the levels of succinate in the cell, which is described in detail in macrophages (M1), that, upon activation, rearrange metabolism so that a certain analogue of succinate, itocanate, can be formed from cis-aconitate [74,75] (see Figure 2); and itocanate has powerful anti-inflammatory potency [76]. Two populations of macrophages are known to be proinflammatory and anti-inflammatory (M1 and M2, respectively), and both populations are present in the tumor microenvironment. Hypoxic changes in the tumor lead to stimulation of the formation of the M2 phenotype, which supports tumor growth and progression. In the cytosol of activated macrophages (M1), there is an increase not only succinate and itaconate, but also citrate, indicating that the Krebs cycle is seriously compromised [77]. Note the high degree of infiltration of macrophages into the tumor; as a result, the mass of macrophages in the tumor can comprise up to 50% of a tumor’s mass [78]. Therefore, in the tumor microenvironment, there is a significant increase in itaconate. The latter blocks succinate dehydrogenase, thereby inducing the oncogenic process due to an increase in the tumor growth-potentiating succinate and itaconate in the microenvironment [79,80]. In addition to retardation of succinate dehydrogenase, inhibition of citrate dehydrogenase is observed in tumor-associated M1 macrophages [77]. We observe an unusual signaling of Krebs cycle metabolites, in particular succinate, in the tumor, unrelated to mitochondrial energetics, in which the tumor microenvironment and in particular macrophages play an important role [81].

**Figure 3:**
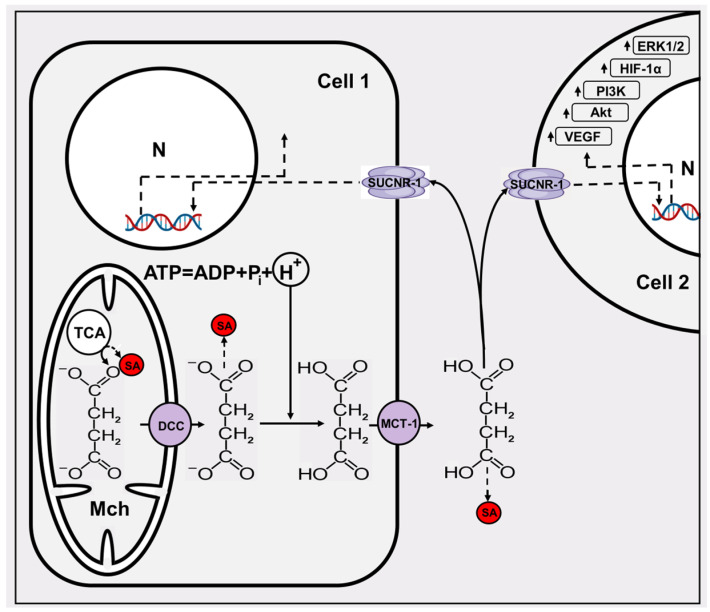
The minimal scheme of succinate signaling. Two cells (1 and 2) are given, one of which, due to several influences, becomes a super producer of succinate. From the mitochondria (Mch), where succinate is primarily formed, it exits into the cytosol through the dicarboxylate carrier DCC. Succinate leaves the cell in a protonated form through the MCT-1 transporter, and in a cell in which the pH is low (hypoxic or cancerous), the probability of succinate protonation is high. Acidification of the cytosol is mainly determined by the ATPase reaction. Extracellular succinate interacts with the SUCNR-1 of both the donor and surrounding cells, triggering expression of several genes in the nucleus (N) and the up arrows near the boxes indicate an increase in their levels. SA, succinylated protein adduct mediated by succinyl CoA formed in Krebs cycle and amino acid metabolism (see the text).

Outside the cell, succinate appears due to its release into the extracellular space using a dicarboxylate transporter located in the plasmalemma (MCT-1). Succinate released from cells may have beneficial effects due to its transfer to cells that are not deficient in oxygen and are quite capable of using it in the succinate dehydrogenase reaction followed by oxidation to water and CO_2_. For example, such a transfer is described for energy communication between the retina, which chronically experiences problems with oxygen delivery to the eye, and oxygen-rich eye cells (retinal pigment epithelium-choroid) [82]. A similar transport, mediated by MCT-1, has been described for muscle tissue, in which, as a result of active muscle work, there is also an accumulation of succinate. The latter, being released from myofibrils, activates non-muscle resident cells such as stromal, endothelial, and satellite cells, causing remodeling of muscle cells after interaction with the succinate receptor SUCNR-1 [83]. The value of SUCNR-1 which has the common name G protein coupled receptor 91 (GPCR91), is not limited to its known role in kidney function [84]. Currently, a large body of data has been accumulated on the involvement of GPCR91 in several diseases, including cancer. One of the extremely important pathogenetic factors in oncogenesis, mediated by an increased content of succinate in the blood of cancer patients, is its role in the organization of the tumor environment. There, succinate plays the role of a paracrine factor that enhances the migration of tumor cells and angiogenesis in tumors, that is, to be a promoter of tumor growth and metastasis (reviewed in [85], minimal scheme is given in Figure 3).

One of the important pathogenetic mechanisms induced by succinate is the inhibition of oxidative hydroxylation of HIF-1α, halting the degradation of this important transcription factor. As a result, even under normoxia conditions, HIF-1α is present and activates the transcription of several genes encoding proliferative, metabolic angiogenic, and proinflammatory proteins [86,87]. Extracellular succinate increases HIF-1α expression via the SUCNR-1–PI3 K/Akt signaling pathway [88].

Elevated intracellular and extracellular succinate levels can potentially cause serious changes in proteins due to their succinylation. Protein succinylation, which is reduced to the addition of a succinyl residue to the ε-group of lysine, is a powerful modulator of protein activity (e.g., see [89,90,91]). The structure of modified protein dramatically changes due to a change in the isoelectric point of a protein (instead of one intrinsic positive charge, the lysine residue acquires a negative charge). Succinyl-CoA, formed as a result of the Krebs cycle or amino acid metabolism, serves as a donor for such posttranslational modification, which can be both enzymatic and non-enzymatic ([92], reviewed in [93]).

Thus, the appearance of succinate in the circulation is not only an indicator of pathology, in particular mediated ischemia [94], but also an initiator of the development of several pathologies.

A reasonable conclusion to tackle the cancer suggests itself: it is necessary to limit the release of succinate from ischemic, in particular cancerous, cells. In principle, based on their mechanism of succinate travel from formation to appearance in the bloodstream, it is first necessary to understand the inevitability of succinate formation in ischemic areas, particularly inside solid tumors. However, it is possible to limit the potential adverse effect of high levels of intracellular succinate. It can be achieved by reducing the generation of ROS caused by the reverse transport of electrons along the respiratory chain by adding so-called “mild” uncouplers. It is well known that this extensive class of agents significantly reduces the generation of ROS in mitochondria, while its therapeutic effect in oncology is understood [95,96,97,98,99,100,101].

Second, as indicated above, succinate leaves the cell in a largely protonated state, which is caused by a lower pH value in the tumor cell [102]. Once again, we note that this acidification is largely explained by the imbalance between ATP generation and its use, given that the ATPase reaction is accompanied by the release of a proton, while the lower the ATP level (higher the ADP level) the greater the acidification (see the reaction scheme in Figure 3). Once again, it should be noted that the acidification of the cytosol can in no way be explained by switching metabolism to the glycolytic pathway of energy formation (see explanation in [103]). To prevent undesirable acidification and the inability to reduce energy levels, it is necessary to increase the buffering capacity of the cytosol. It is possible to normalize the cytoplasmic pH in a tumor cell by, for example, adding dipeptides [104].

The third line of anti-cancer/anti-succinate defense may be in limiting the transport of succinate into the extracellular space by inhibiting MCT-1. Recent publications provide an example of the use of a specific inhibitor of this carrier (AZD3965), demonstrating the positive effects of currently ongoing clinical trials [105,106].

Fourth, the succinate receptor SUCNR-1 (GPCR91) may be another target for limiting the regulation of cancer cell proliferation. So far, the possibility of suppressing this receptor has not been widely discussed in the literature, but indirect actions indicate that this is a possibility. When analyzing the suppressive effect of exosomes derived from mesenchymal stem cells on metastasis of rectal cancer tumor cells, it was noted that these exosomes contain microRNA-1827 (mir-1827), which inhibits SUCNR-1 [107].

## 5. Mammalian Mitochondrial (Peripheral) Benzodiazepine Receptor

**Figure 4:**
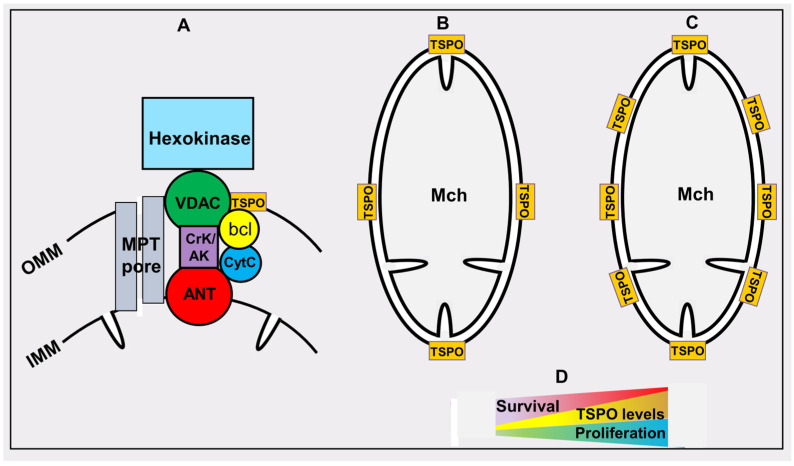
Tryptophan-rich-sensory protein (TSPO)/peripheral (mitochondrial) benzodiazepine receptor. OMM and IMM, outer and inner membranes of mitochondria (Mch), respectively. (**A**) the architecture of the TSPO environment: voltage dependent anion channel (VDAC); creatine kinase/adenylate kinase (CrK/AK); adenine nucleotide transporter (ANT), bcl-2 and bcl-xl (bcl); mitochondrial permeability transition pore (MPT pore). (**B**,**C**) relative levels of TSPO in normal and highly proliferating cells correspondingly. (**D**) the relationship between TSPO levels and cancer cell proliferation and survival of cancer patients.

One of the intriguing components of mitochondria is a specific protein, strikingly similar to that which in modern nomenclature is listed as the tryptophan-rich-sensory protein (TSPO [108]) in *Rhodobacter sphaeroides*, or the protein involved in carotenoid biosynthesis (CrtK) [109] in *Rhodobacter capsulatus*. Currently, all these different but related proteins have a common name, TSPO. This protein, which has a small mass (about 18 kDa), is localized within the outer mitochondrial membrane. Previously known as the peripheral benzodiazepine receptor (PBR), it functions as a counterweight to the central benzodiazepine receptor localized in the brain [110]. Its mitochondrial protein partners are at least two proteins, with one also belonging to the outer membrane, a voltage-dependent anionic channel (VDAC), and an inner membrane protein, a translocator of adenine nucleotides (ANT) [111] (Figure 4). Note that all three components belong to the mitochondrial structures known as contact sites; these have a very interesting history of elucidation due to their participation in the organization of nonspecific permeability of mitochondria, taking part in cell death [112,113,114,115,116]. The identical sensitivity of ion channel activity formed by contact sites to PBR ligands and inhibitors of mitochondrial nonspecific permeability (mitochondrial permeability transition pore, MPT) suggests that mitochondrial permeability is the functional state of PBR [117,118]. However, it should be noted that there is currently no clear understanding of the structural organization of MPT in the scientific community, although the model of involvement of ATP synthase (in the form of a dimer) in the organization of MPT currently prevails [119]. There is a strong and reasonable objection to this model [120], which states that the phenomenon of mitochondrial permeability transition does exist, but that the material base responsible for this phenomenon is unclear.

Some synergistic effect between TSPO and its partners on the mitochondrial contact site can be assumed. As with TSPO, a direct association of the level of VDAC and the degree of cancer aggressiveness is known. VDAC is a predictive biomarker for some types of cancer [121,122,123], although this association is VDAC isoform-specific. We also note that other partner/partners on the contact site, namely bcl-2/bcl-xL, are known to ensure cell longevity [124,125]. Another TSPO partner found at the mitochondrial contact site is hexokinase [114,115,126,127], a well-known hub between oxidative and anaerobic energetics pathways [16,128]. Many functions are attributed to the TSPO, which has received the name polytopic protein due to fact that PBR ligands affect several processes in the cell. Given its increased distribution in steroidogenic tissues and secretory glands, PBR was credited with participating in the synthesis of steroids (which are known to be synthesized in mitochondria [129]) by using cholesterol transported from cytosol to the mitochondrial matrix [130]. In addition, it was proposed that PBR plays a role in the mitochondrial transport of porphyrins, including protoporphyrin IX and heme [131], which is also synthetized in mitochondria [1]. In addition, it plays some role in immune responses [132,133], energy metabolism [134,135], production of reactive oxygen species [136], apoptosis [137], and cell proliferation [138]. The idea that the PBR serves as an oxygen sensor is quite intriguing. In bacterial cells, CrtK serves as an oxygen sensor that regulates the expression of several genes depending on the oxygen tension. In bacteria with the corresponding gene knocked out, the introduction of the human PBR gene restored the lost functions of oxygen sensing [139].

The relevance of TSPO to cancer is very high, although the reason for this is incomprehensible. The levels of TSPO mRNA, as well as the protein itself, in cancer cells are usually much higher than in normal cells, indicating that transcription of TSPO increases in hyperplastic tissue [140]. Increased TSPO level was observed in several types of neoplastic tissue, including breast cancer [141,142], colon cancer [143,144], brain cancer [145,146], prostate cancer [147], esophageal cancer [148], endometrial carcinomas [149], ovarian cancer [150], and several other types of cancer cells. TSPO gene amplification has been witnessed in human breast cancer cell lines [151] and metastases [152]. In addition, there was a definite association between the level of aggressivity of cancer and the levels of TSPO in these cancer cells; this suggests that levels of TSPO can be a good indicator of an aggressive phenotype in several cancers [153]. Generally speaking, high levels of TSPO in malignant tumors were found to be associated with a worse prognosis [154]. The normalization of PBR to the mass of mitochondria did not lead to a definite correlation between these two parameters [155]; this allows us to present the scheme shown in Figure 4. The content of this protein in individual mitochondria increases regardless of the increase in the mitochondria mass, which often occurs in a number of tumors. The abundance of TSPO in cancer cells has been used in positron emission tomography (PET)-imaging with TSPO-specific ligands to detect tumor cells [156,157,158,159].

An abundance of TSPO in cancer cells is used in photodynamic therapy after recruitment of photo-sensitive TSPO ligands [158,159]. The same characteristic feature of an abundance of TSPO in aggressive tumors was exploited for practical purposes by using TSPO ligands as anti-cancer drugs [160,161,162]. In this sense, the TSPO ligands can also be considered as mitocans. However, the practical use of TSPO ligands is beyond the fundamental knowledge of the relevance of this protein to oncogenic transformations [163], which requires an immediate solution.

The above-mentioned role of TSPO in the transport of cholesterol in the mitochondria may play one of the key roles in tumorogenesis, given the significant amount of data on the participation of this lipid in tumor growth [164,165,166]. There are noticeable changes in cholesterol homeostasis in tumor cells; this is accompanied by an increase in cellular cholesterol, which is formed in mitochondria. This is the result of the activation of genes involved in the synthesis of cholesterol and an increase in its absorption by cells mediated by low-density lipoprotein receptors and abnormal cholesterol metabolism (reviewed in [166]). Ultimately, one potential anti-cancer strategy is the use of substances that reduce the cholesterol content in the tumor (reviewed in [167]).

## 6. High Retention of Cationic Dyes in Cancer Cells

We will briefly consider a rather old topic that is not very widely discussed in modern research. With a full understanding of the mechanism underlying an important previously discovered phenomenon, it would be possible to obtain several practical aspects in the anti-cancer defense. The starting point of this line of research is the discovery of the phenomenon of retention of cationic dyes in tumor tissues [168]. This work came out of a laboratory led by Lan Bo Chen, who was the first to propose the use of rhodamine 123 for imaging mitochondria in cells [169]. This gave a powerful impetus to determine the direction of non-invasive or minimally invasive vital detection of mitochondria, implemented in the form of modern development and development of chemical agents carrying a delocalized positive charge. The intensity of fluorescence emitted by these probes under ideal conditions reveals the magnitude of the mitochondrial membrane potential; it is a vital indicator of the functional state of mitochondria, i.e., the higher the fluorescence of these probes, the higher the transmembrane potential is on the inner mitochondrial membrane [170]. Combining two studies published in 1980 and in 1982 [168,169], when the latter revealed the unusual property that retention of cationic dye in tumor cells was significantly higher than in related non-cancer cells, it was concluded that the mitochondrial membrane potential is higher in tumor cells than in non-tumor cells; this argument was used by the same laboratory in later publications [171,172,173]. The main argument for this statement was based on the data from experiments in which fluorescence remained at a significant level after one day of incubation after loading cancer cells with rhodamine 123 and placing them in a rhodamine-free medium, while it quickly disappeared from normal cells. However, this interpretation does not consider the mechanism of the accumulation of the amphiphilic cation probe, which must be distributed in full accordance with the value of ΔΨ and, being a slow or “redistributive” probe, reaches a steady state quickly in the absence of binding to matrix structures or changes in permeability properties [174]. Therefore, the interpretation of the increase in fluorescence accumulated in the mitochondria of the probe as a reflection of the increased membrane potential has been argued [175].

In general, the mechanism of retention of various chemicals in tumor cells and/or tissues remains unclear even in the historically more developed branch of medicine as photodynamic therapy, which in practical terms has been used for quite a long time. Several photosensitizers have been developed, and each group has its own mechanism of specific accumulation in different cellular compartments. Mitochondrial photosensitizers normally carry a total positive charge, allowing them to accumulate in the organelles [176]. However, there are other mechanisms that allow photosensitizers to accumulate in mitochondria by binding to protein components (for example, with the above-mentioned TSPO [177] or other mitochondrial proteins [178,179,180]), as well as with lipids with the characteristic mitochondrial lipid, cardiolipin, being the mechanism by which nonyl acridine orange binds to mitochondria [181,182].

**Figure 5:**
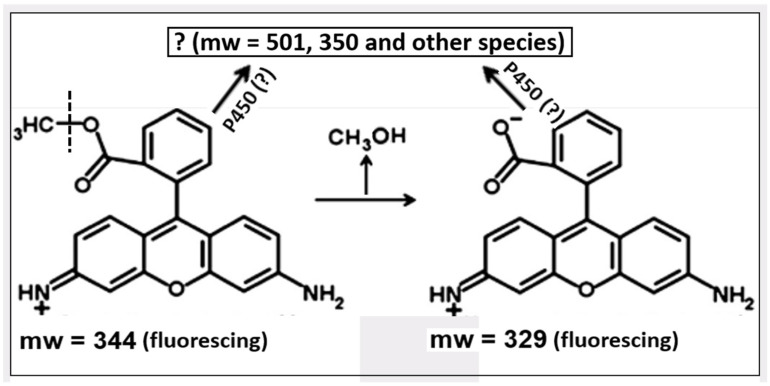
Possible modifications of rhodamine 123. Apart from deesterification of the probe associated with the release of methyl alcohol, several modifications of the initial probe and its product can take place, possibly with involvement of cytochrome P450 (for details, see [175]).

The development of mitochondrial-directed photosensitizers continues, and, considering its long history, it has an obvious future [183,184,185,186,187,188,189]. Unfortunately, the mechanisms of specific accumulation and retention in tumor tissues remain unclear, and their understanding lags behind the available practical application. However, recent studies have outlined some trends in determining future directions in the study of the possibility of using mitochondrial-directed compounds as an anti-cancer strategy [175]. It was shown that the previously detected retention of rhodamine 123 in tumor cells is associated with various modifications of probe molecules (including those with participation of cytochrome P450) with the possibility of the formation of forms whose exit from mitochondria is difficult or associated with binding to mitochondrial components [190,191,192]. As one of the possibilities, the deesterification of the rhodamine molecule with the formation of a zwitter ion (rhodamine 110), whose permeability through phospholipid membranes is significantly lower than that of the parental cation [193], may be one of the reasons for the long retention of the fluorescent molecule (see Figure 5; note that the fluorescent properties of rhodamines 110 and 123 are the same). This indicates the need to study enzymes that modify the xenobiotic molecule in such a way that it is retained in the tissue and maintains its anti-tumor properties [194,195], thus contributing to the accelerated death of a cancer cell.

As we noted above, the distinctive anchoring of several molecules in the mitochondria of tumors may be due to difficulty in exiting because of thermodynamic limitations (for example, if a hydrophilic/amphiphilic agent carries a total negative charge, as a result of which the membrane potential (minus inside) retains this agent in the mitochondrial matrix). The second possibility is a stronger (electrostatic and maybe even covalent) binding of mitochondrial components with the characteristic mitochondrial lipid, cardiolipin. To better visualize mitochondria, an approach has been applied in recent work that may well be used to solve the question of the mechanism and practical use of lipid mitochondrial probes. Two-stage staining of mitochondria was applied, first with one cardiolipin-specific probe (a derivative of nonyl acridine orange), which was subsequently conjugated with another, and second with a fluorescent agent [196]. Given the high fluorescent stability of this system, and consequently the high quantum yield of fluorescence of the proposed agents, it is unlikely to be used in photodynamic therapy. However, it is also possible to build a chemical system based on this same principle in which an agent anchored with mitochondria is conjugated with a toxic substance, ultimately killing mitochondria and subsequently the host cell.

## 7. Brief Conclusions

To date, a large amount of data has accumulated regarding the special role of mitochondria not only in the occurrence of cancer but also about the possibility of applying a strategy for the treatment of this disease. It is based on knowledge of known biochemical mechanisms whose activity changes during oncogenic transformation and structural and functional features of mitochondrial energetics and other mechanisms indirectly related to energetics [196,197,198,199]. The three lines of enquiry presented in the review, which can be further investigated and used to tackle cancer, are too far away from being comprehensive in the fight against this pathology, the distinctive feature of which is the exceptional heterogeneity of cellular metabolism. Given the limited possibilities of this review, we only mentioned the switch in several cancer cells from predominantly oxidative to anaerobic energy production, pointing out the possible elements involved in this switch. The energy metabolism of various cells, in particular cancer cells, can use a combination of these two basal energetics, the so-called aerobic glycolysis (e.g., [200,201]), thus producing energy depending on the environment with different availability of oxygen and substrates. In any case, the main goal of a cancer cell is to perform its own proliferation in any way possible, which requires energy and building materials. In addition to the reversal of the succinate dehydrogenase reaction in a cancer cell discussed above, another pathway has been demonstrated in a recent study, formally consisting in the reversal of another partial reaction (namely malate dehydrogenase) in the Krebs cycle. As a result, NAD^+^ is formed, which drives reactions that promote the entry of substrates necessary for building new cells [202].

The three pathways selected in this review are rarely reviewed aspects that can help determine the targets and additional elements of anticancer strategy, thereby stimulating general interest to the role of mitochondria.
